# Collective Motion of Cells Mediates Segregation and Pattern Formation in Co-Cultures

**DOI:** 10.1371/journal.pone.0031711

**Published:** 2012-02-16

**Authors:** Előd Méhes, Enys Mones, Valéria Németh, Tamás Vicsek

**Affiliations:** 1 Department of Biological Physics, Eötvös University, Budapest, Hungary; 2 Statistical and Biological Physics Research Group of the Hungarian Academy of Sciences, Budapest, Hungary; Hungarian Academy of Sciences, Hungary

## Abstract

Pattern formation by segregation of cell types is an important process during embryonic development. We show that an experimentally yet unexplored mechanism based on collective motility of segregating cells enhances the effects of known pattern formation mechanisms such as differential adhesion, mechanochemical interactions or cell migration directed by morphogens. To study in vitro cell segregation we use time-lapse videomicroscopy and quantitative analysis of the main features of the motion of individual cells or groups. Our observations have been extensive, typically involving the investigation of the development of patterns containing up to 200,000 cells. By either comparing keratocyte types with different collective motility characteristics or increasing cells' directional persistence by the inhibition of Rac1 GTP-ase we demonstrate that enhanced collective cell motility results in faster cell segregation leading to the formation of more extensive patterns. The growth of the characteristic scale of patterns generally follows an algebraic scaling law with exponent values up to 0.74 in the presence of collective motion, compared to significantly smaller exponents in case of diffusive motion.

## Introduction

Understanding pattern formation in various fields of life and especially in the morphogenetic events taking place in developing embryos has always been a subject of interest in biology. Several models have been proposed to explain these events. The Turing theory argues that diffusible morphogens are produced and diffuse forming a heterogeneous steady-state spatial prepattern and thus different local morphogen concentrations react with cells to differentiate them [1]. This theory based on the reaction-diffusion assumption has been widely applied for several pattern formation events although not many morphogens have been identified as yet. The model systems based on this theory are generally very sensitive to variations in parameters.

The Murray-Oster mechanochemical model of pattern formation is based on the view that local cellular forces act on the viscoelastic extracellular matrix and other cells thus creating spatial heterogeneity leading to changes in pattern and form [2]. If combined with the Turing prepattern assumption the local interactions of the mechanochemical approach can result in a more robust morphogenetic mechanism that also allows for local self-correction. Pattern formation in real embryonic development is indeed a very stable process which should be resistant to small perturbations during the patterning process. The differential adhesion hypothesis argues that difference in the adhesion properties is a key component of cellular pattern formation by segregation [3], [4]. Experimental observations with avian and fish embryonic cells have demonstrated that pattern formation is greatly influenced by the cells' surface tension resulting from their different adhesion properties [5], [6].

Pattern formation in embryonic morphogenesis is a fast process in several species, however the reason for such speed is a long unsolved problem suggesting that several already known but not fully explored mechanisms should act in a synergistic way to accelerate such processes.

We demonstrate the existence of a mechanism for pattern formation based on segregation (also referred to as sorting-out) in which cell migration is a key component in addition to differential adhesion. Cell migration is often seen as persistent random-walk in 2-dimensional cell culture. It is characterized by cell velocity and directional persistence, i.e. how long the cells tend to move linearly before changing direction. If directional persistence is high, groups of cells are more likely to move in a correlated fashion. This correlated or collective migration (also referred to as cohort migration or sheet migration) is further enhanced by adhesive bonds between individual cells. In computer simulations where cell adhesion strength, cell velocity and correlation between cell velocities are adjustable parameters it has been shown that a population of two initially mixed cell types gradually segregates in homogeneous clusters in a self-propelled manner [7]. Such simulated cell segregation is enhanced even by a slight increase in the correlation of cells' motion, whilst the time dependence of the progression of the process turns from slow logarithmic to faster algebraic scaling.

We argue that collective migration resulting from high directional persistence of individual cells facilitates and accelerates the formation of segregated clusters from a mix of cell types. To support this notion we present in vitro experiments and analyses with mixed cell types where cell velocity and directional persistence are varied or altered by drugs. These experimental variations result in characteristic differences in both the speed and extent of cluster formation by self-propelled segregation. Increased collective cell migration, characterized by larger directional persistence, leads to faster and more extensive cell segregation yielding larger segregated clusters. We demonstrate that the growth of the characteristic scale of the segregated patterns follows an algebraic scaling law with high exponent values showing the accelerating effect of collective cell motion on the segregation process, compared to the lower exponent values resulting from simulations in the absence of collective motion [8]. Our experimental data are from patterns formed in co-culture containing up to 200,000 cells, with overall size comparable to a fish embryo. These data support the predictions of simulations of segregation with correlated cell motion, typically involving a few thousand cells [7].

Analogies can also be found between in vitro experiments with mixed 2D cell cultures and cell migration related pattern formation during embryonic development. Exploring the impact of collective migration on pattern formation of cells in 2D culture may also provide new ways for pattern formation in tissue engineering.

## Results

### Directional persistence is a key factor in collective migration

**Figure 1:**
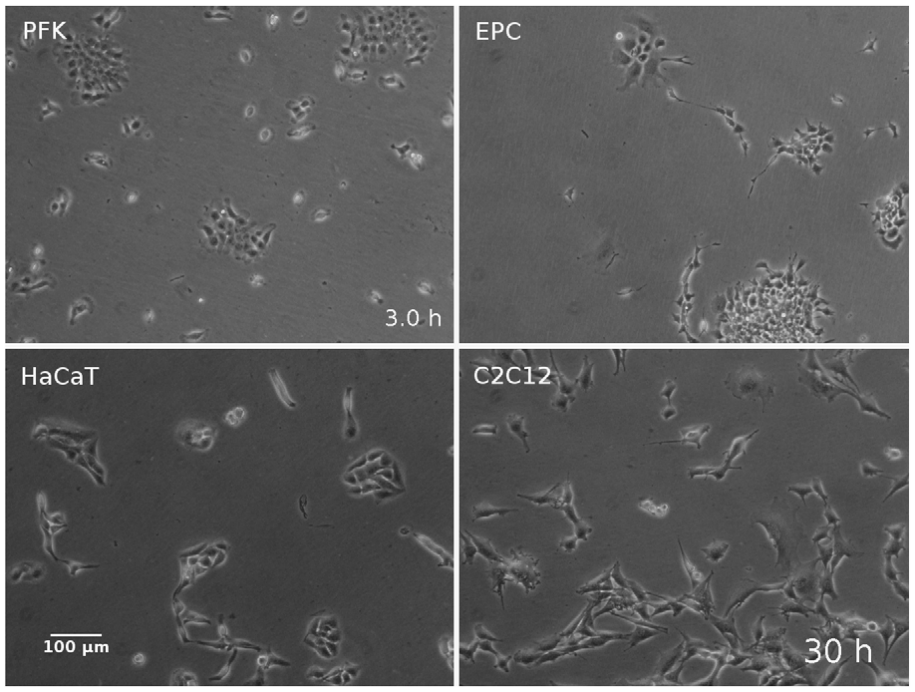
Morphology and patterns of the cell types studied. Phase contrast images of the cell types studied: primary goldfish keratocytes (PFK), fish keratocyte cell line (EPC), human keratocyte cell line (HaCaT) and mouse myoblast cell line (C2C12). See also Video S1.

Collective cell migration is characterized by lateral association of individual cells migrating in a correlated way with high directional persistence. To study the impact of collective migration on cell segregation phenomena we used mixed monolayer co-cultures of keratocyte types from various species and video-imaged the formation of segregated cell clusters. We used primary keratocytes from goldfish, EPC fish keratocyte line (originally isolated from fathead minnow) and HaCaT, a human keratocyte line. These keratocyte types can form clusters in a density dependent way and are able to migrate collectively with inherently different parameters of correlated motion. For comparison we also tested C2C12, a mouse myoblast cell line, as these cells tend to migrate as single cells without clustering even at high cell density (Fig. 1).

**Figure 2:**
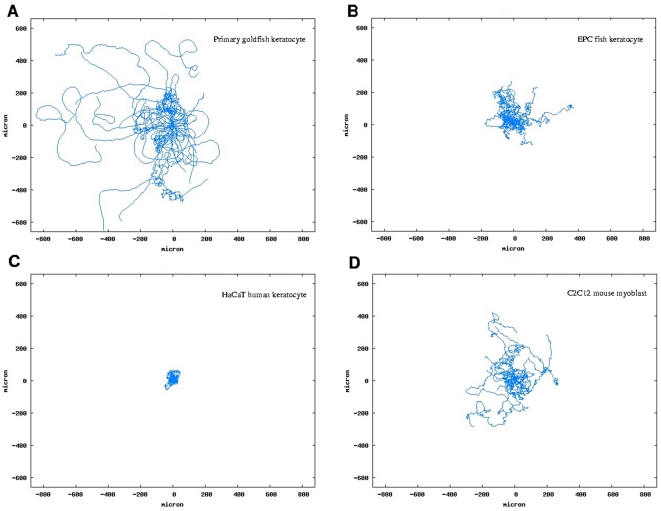
Typical trajectories of the cell types studied. Superposed trajectories of 20 randomly selected single cells from each cell type studied: primary goldfish keratocytes (A), EPC fish keratocytes (B), HaCaT human keratocytes (C) and C2C12 mouse myoblasts (D). Trajectories from 50 hours of observation are shown for all panels except for panel A where 6 hours were observed for similar trajectory lengths due to high velocity of primary goldfish keratocytes. Trajectories are superposed to start from the same origin for better comparison. Note the differences in the curves of typical cell trajectories indicating differences in directional persistence.

The cell types studied have different inherent motility characteristics: they differ in velocity and directional persistence of cell migration. Directional persistence arises from the cell's ability to maintain front-rear polarity during migration. Migrating cells' directional persistence is characterized by their persistence length and persistence time, calculated on the basis of the Orstein-Uhlenbeck model [9] by parametric fitting of Fürth's formula [10] to the empirical displacement(t) curve computed from tracked cell trajectories. The displacement(t) curve shows the average displacements of cells in all possible time-frames of observation. Persistence length is the average distance that a cell tends to migrate linearly without changes in direction and it corresponds to the initial linear segment of the displacement(t) curve. Cell velocity and directional persistence data are summarized in Table 1 for single cells in low density cultures of various cell types and typical trajectories are compared in Figure 2 (see also Video S1). It is seen that cell types migrating more collectively, such as primary goldfish keratocytes, tend to have larger persistence lengths, which is a suitable quantity for characterization of collective migration.

**Table 1:** Summary of motility data of cell types studied.

Cell type	PFK*	EPC	HaCaT	C2C12
Persistent speed (µm/h)	558±28	29±1.6	46±2.9	25±2.1
Diffusion coefficient (µm^2^/min)	132±1.4	1.61±0.01	1.29±0.01	5.0±0.02
Persistence time (min)	3.0±0.2	13.6±0.4	4.4±0.3	57.5±1.3
Persistence length (µm)	24.3±0.7	5.7±0.1	2.9±0.1	20.6±0.2
Average velocity (µm/h)	501±17	30.1±0.8	34±0.6	35±0.5
Time resolution of tracking (min)	1	10	10	10

Cell motility data were calculated from tracked trajectories of ∼50 cells from each cell type at low cell density.

*PFK: primary goldfish keratocyte.

**Figure 3:**
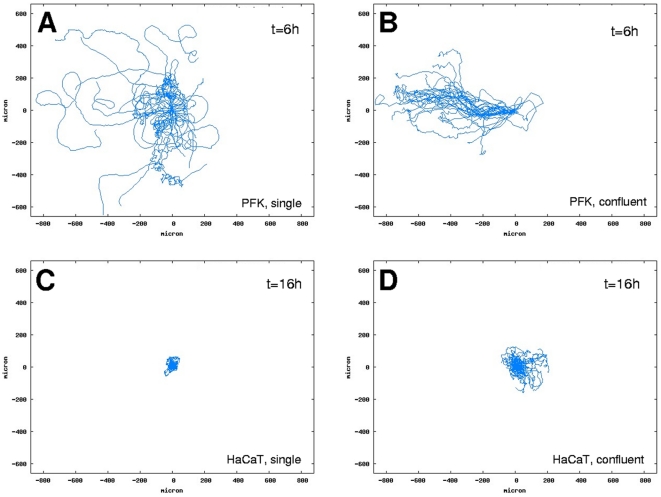
Comparison of trajectories at different cell densities. Superposed trajectories of primary goldfish keratocytes (PFK) (A and B) and HaCaT keratocytes (C and D) migrating either as single cells or in clusters for durations t = 6 h (A and B) or t = 16 h (C and D). Trajectories of 30 randomly selected cells are shown in each panel. Note the increase in linear trajectories in panels B and D compared to panels A and C.

**Figure 4:**
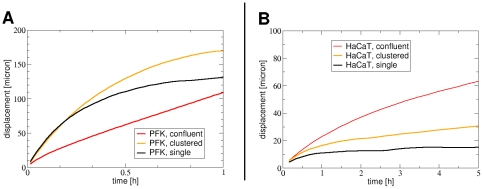
Comparison of cell motility at various cell densities. Empirical distance(t) curves for primary goldfish keratocytes (PFK) (A) and HaCaT keratocytes (B) at different cell densities. Data are from trajectories of cells migrating as singles or in clusters or in confluent monolayer. Note the increase in the linear segment of the red curves, corresponding to highest cell densities, compared to other curves.

Collective cell migration in 2-dimensional cultures is a density-dependent phenomenon. Beyond a critical density [11] each keratocyte type studied tends to form clusters of laterally attached cells. Clustering changes the directional persistence and speed of cells migrating in the cluster (Fig. 3). Directional persistence is increased and cell velocity is decreased upon cluster formation in all keratocytes (Fig. 4, Table 2). Highest directional persistence is characteristic of primary goldfish keratocytes either as single cells or cell clusters. This cell type can form large clusters capable of collectively migrating with high directional persistence over large distances compared to cell size (Video S2). Large increase is seen in the persistence length of HaCaT keratocytes along with an increase in cell density resulting in cell streaming within the monolayer: a form of collective migration similar to what is modeled for endothelial cells [12] (Video S3). The ability of a given cell type to form clusters by lateral cell attachment is required for collective cell migration, which originates from individual cells' ability to maintain their front-rear polarity during migration and synchronize it with others. The ‘collectiveness’ of migration is enhanced if the persistence length of migrating cells is larger: the larger their persistence length, the more correlated these cells' migration becomes (Fig. 4).

**Table 2:** Comparison of cell motility and cell pattern.

Cell type	PFK*			EPC		HaCaT		
Pattern	Single	clustered	confluent	single	clustered	single	clustered	confluent
Speed (µm/h)	623±36	511±29	105±7	35±1.8	21±2.4	63±15.4	46±2.9	32±1.4
Diff. coeff. (µm^2^/min)	116±1.5	158±1.7	134±0.7	1.7±0.01	1.6±0.01	0.6±0.01	1.29±0.01	3.6±0.01
Persistence time (min)	2.2±0.2	4.3±0.2	87.6±1.3	9.7±0.3	26.8±1.2	1.2±0.5	4.4±0.3	25.8±0.4
Persistence length (µm)	19.2±0.8	31.7±0.9	131.5±1	4.9±0.1	8±0.2	1±0.2	2.9±0.1	11.7±0.1

Cell motility data were calculated from tracked trajectories of cell populations in 3 typical densities for each cell type. Note the increase in persistence length for clustered cells in all cell types.

*PFK: primary goldfish keratocyte.

### Collective migration facilitates cell segregation

We utilized the inherent motility characteristics of the cell types studied to test the impact of collective migration, characterized by cells' directional persistence, on the segregation of cell types in co-cultures where two cell types were initially mixed. The basic motive of spontaneous cell segregation in such a system is differential adhesion of the participating cell types. If homotypic adhesion strength (i.e. adhesion between cells of the same cell type) is larger than heterotypic adhesion strength (i.e. adhesion between two different cell types) this difference can be the adhesion-based driving force for spontaneous cell segregation here.

Although we did not quantitatively study cell adhesion between pairs of the studied cell types it is observed that none of these cell types tends to spontaneously mix with or attach to the other cell type, instead they tend to segregate (Video S4). Therefore heterotypic adhesion in our system is considered to be very low compared to homotypic adhesion. The characteristics of in vitro patterns observed and the speed of their formation heavily depend on the following properties of cells and cell groups: i) their relative density, ii) relative speed, iii) persistence lengths during individual or group migration.

We thoroughly mixed pairs of cell types after labeling each cell type either red or green using fluorescent cell tracking dyes as indicated for the specific case. Confluent mixed co-cultures were made from keratocyte types: EPC+primary goldfish keratocytes (Video S5) and EPC+HaCaT (Video S6). For comparison mixed cultures were also made from C2C12+HaCaT (Video S7). Equal ratio of the cell types (1∶1) was provided in terms of area covered by each cell type, taking into account the inherent differences in cell size among the cell types studied. This ratio was maintained as cell death and cell division were negligible in the analyzed period of experiments. Primary goldfish keratocytes do not divide in such cultures whereas the observed doubling times of EPC, HaCaT or C2C12 cells in confluent monolayers exceeded the duration of experiments. The mixed co-cultures were observed by videomicroscopy for up to 48 hours and the size of emerging homogeneous clusters of the participating cell types were continuously monitored. Segregation of any two cell types were monitored by consecutively calculating two-point correlation functions for each cell type then extracting the correlation lengths which correspond to the diameters of emerging homotypic cell clusters (see Materials and methods). Cell cluster sizes were also measured by counting the area (pixels) of individual cell clusters by image analysis. This in vitro system allows the observation of unbiased cell segregation for about 25 hours since beyond this time scale a few perturbations start to have effect on the data: e.g. cell division (for cell lines only), a minor but increasing ratio of cell deaths and the onset of new modes of collective behavior such as jamming [13]. For these reasons we have numerically analyzed the data obtained for the first 17 hours of the pattern formation process in most cases.

**Figure 5:**
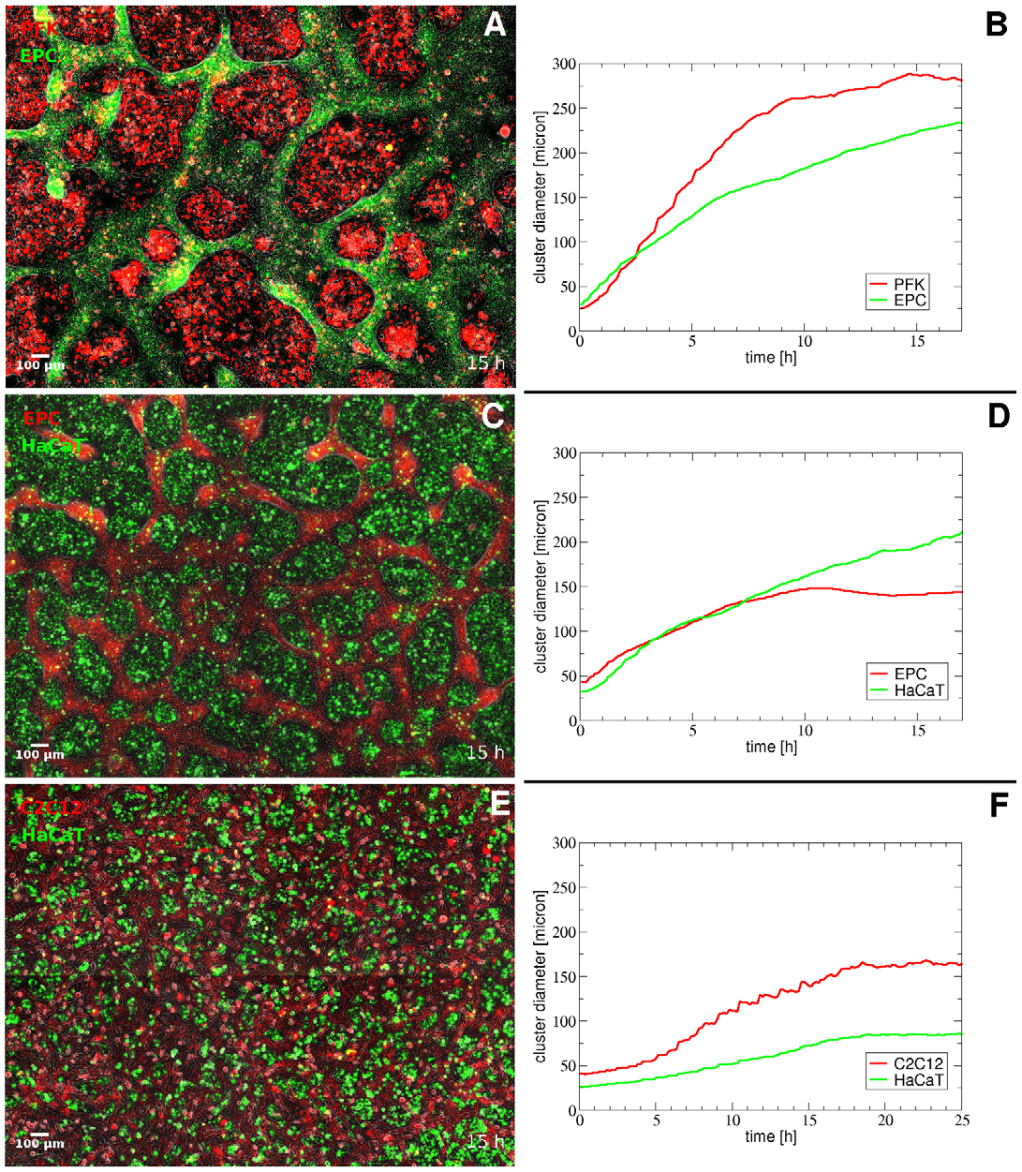
Comparison of co-culture cell clusters and cluster formation dynamics. Left: merged fluorescent and phase contrast images of representative segregating co-cultures of primary goldfish keratocytes (PFK) + EPC keratocytes (A) or EPC keratocytes + HaCaT keratocytes (C) or C2C12 myoblasts + HaCaT keratocytes (E) taken when cluster sizes have reached maximum. Right: temporal changes in segregated cell cluster sizes calculated by two-point correlation method. Note the considerable differences in cluster sizes between panels A and C. See also Video S5, S6 and S7 of the same fields (A, C and E).

**Figure 6:**
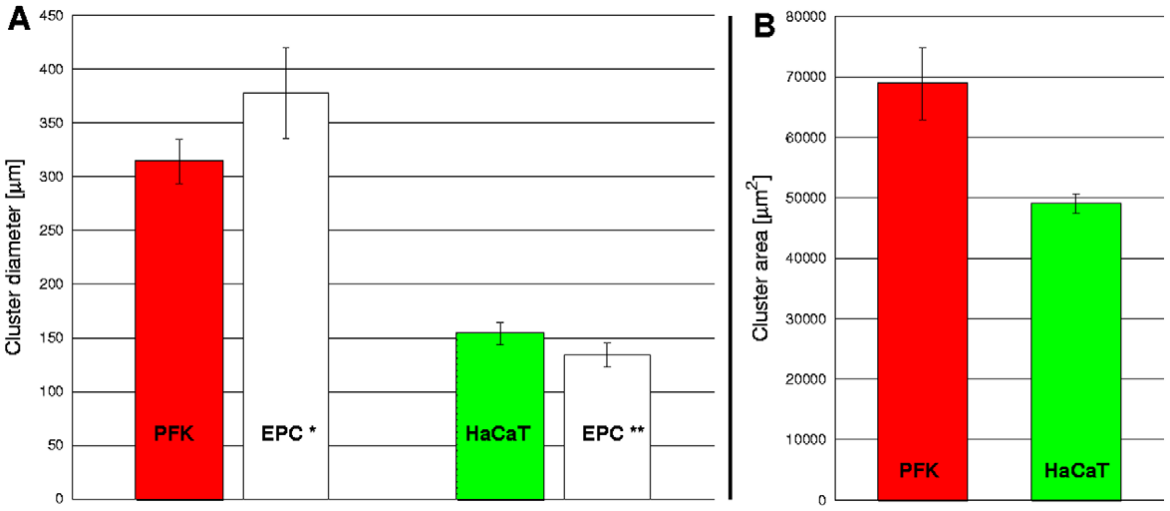
Comparison of cell cluster formation data: diameter and area. Final segregated cell cluster diameters (A) and cluster areas (B) in primary goldfish keratocyte (PFK) + EPC co-cultures (n = 7) or HaCaT + EPC co-cultures (n = 3). Error bars represent SEM. Note the difference between PFK and HaCaT cluster diameters and areas and also the difference between the diameters of EPC clusters in the two co-culture settings (EPC* and EPC**).

Cluster formation is strongly influenced by the motility characteristics of the participating cell types: their relative speed and persistence length. Figure 5 shows the result and dynamism of cluster formation in various mixed cultures. Largest clusters are formed and cluster formation is the fastest by primary goldfish keratocytes that are characterized by the largest persistence length and cell velocity, measured in monotype cultures. Segregated cell cluster sizes are much smaller and take longer to form in the co-culture of HaCaT cells, characterized by lower persistence length, and EPC keratocytes. It is also seen that the network of EPC keratocytes is also made of smaller clusters when mixed with HaCaT keratocytes, compared to mixing with primary fish keratocytes, indicating that cluster formation is also influenced by the motility of the other cell type of the pair. The smallest clusters are observed and take the longest time to form in the co-culture of HaCaT cells with C2C12 myoblasts, which do not migrate in a collective way. Figure 6 shows the comparison of average final cluster sizes in co-cultures of EPC keratocytes with either primary goldfish keratocytes or HaCaT keratocytes. Cluster sizes are characterized by both their average diameters and their average area, calculated with different methods while the error bars represent the standard error of the mean (SEM) in all diagrams.

### Increasing the directional persistence of migration enhances cell segregation

To further investigate the impact of directional persistence on segregation and cluster formation we changed the directional persistence of cells using NSC-23766: a specific inhibitor of Rac1 activity [14]. Rac1 GTP-ase regulates the polymerization of actin into network, driving the formation of the lamellipodium of migrating cells. Lowering the activity of Rac1 by RNA interference method is known to decrease the frequency of new peripheral lamellipodium formation, required for directional changes and this eventually leads to more persistent cell migration in several cell types including epithelial cells, fibroblasts and glioblastoma cells [15].

We tested various concentrations of this Rac1 inhibitor on all cell types studied and found that primary goldfish keratocytes responded to low concentrations (30 µM) of NSC-23766 by altering their motility whereas other cell types were responsive to much higher concentrations (over 150 µM) of the drug (data not shown). Therefore we used primary goldfish keratocytes and EPC keratocytes for tests with Rac1 inhibitor. Higher Rac1 inhibitor concentration (60 µM) in mixed cultures caused prompt rounding up and eventual death for primary goldfish keratocytes but did not affect the motility or morphology of EPC keratocytes.

Primary goldfish keratocytes at various densities responded to low concentrations (30 µM) of NSC-23766 by increasing their persistence length while there seemed no change in their morphology or adhesion. As the persistence increasing effect depended on cell density, we measured persistence lengths both in low density cultures of solitary goldfish keratocytes and in high density mixed cultures of goldfish keratocytes and EPC cells. In both settings partial Rac1 inhibition resulted in considerable increase in persistence length (Table 3). Velocity of goldfish keratocytes was mostly unaffected by Rac1 inhibition in low density pure cultures but increased in high density cultures mixed with EPC keratocytes.

**Table 3:** Comparison of cell motility and Rac1 inhibition.

Cell type	Primary goldfish keratocytes			
Density	Low density*		High density, mixed**	
30 µM Rac1 inhibitor NSC-23766	No	Yes	No	Yes
Speed (µm/h)	782±48	744±55	436±43	622±42
Diff. coeff. (µm^2^/min)	111±0.73	168±5.07	18.13±0.38	62.44±1.94
Persistence time (min)	1.30±0.14	2.18±0.21	0.68±0.16	1.16±0.25
Persistence length (µm)	14.6±0.8	23.3±1.2	4.3±0.5	10.3±1.1

Partial inhibition of Rac1 results in characteristic changes in cell motility parameters calculated from tracked trajectories of cells at different densities and culture compositions. Note the increase in persistence length in the presence of Rac1 inhibitor.

*Low density culture of single cells.

**High density mixed co-culture of primary goldfish keratocytes and EPC keratocytes.

**Figure 7:**
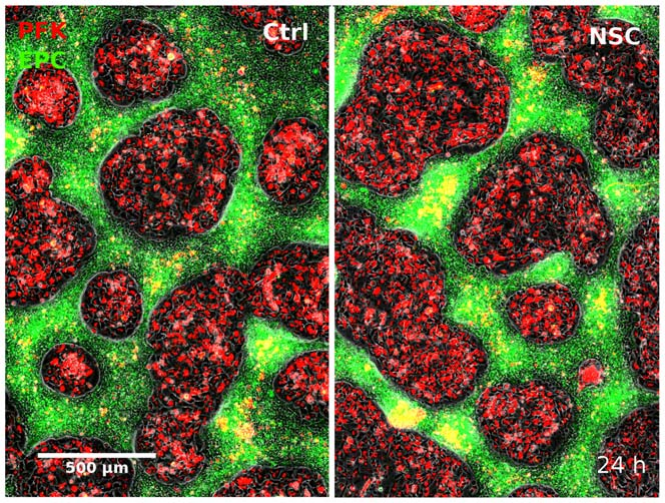
Comparison of cell cluster formation without/with Rac1 inhibition. Comparison of fields of views showing developing clusters in mixed co-cultures of primary goldfish keratocytes (PFK, red) and EPC fish keratocytes (green) 24 hours after seeding. Untreated culture (A) is compared with the culture treated with 30 uM Rac1 inhibitor NSC-23766 (B). Note the larger red PFK clusters in B. See also Video S8.

**Figure 8:**
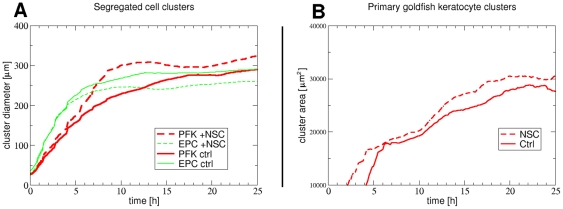
Cell cluster formation dynamics without/with of Rac1 inhibition. Temporal changes in segregated cell cluster diameters measured as two-point correlation lengths (A) and cluster areas (B) for primary goldfish keratocytes (red) and EPC keratocytes (green) in representative fields of views in confluent mixed co-cultures not treated (solid lines) or treated with Rac1 inhibitor NSC-23766 (30 µM) from the beginning (dashed lines). Note the formation of larger primary goldfish keratocyte clusters (dashed red line in A and B) as a result of Rac1 inhibition. See also Video S8.

We used the persistence length increasing effect of the Rac1 inhibitor to increase the collective characteristic of the migration of primary goldfish keratocytes. We simultaneously monitored cell cluster formation in several untreated or NSC-treated confluent mixed cultures of EPC and primary goldfish keratocytes and compared cluster diameters and areas (Figure 7 and Video S8). Fig. 8 shows the dynamics of cluster formation: average cluster diameters were continuously measured by the two-point correlation method as correlation lengths for two representative fields of view. Average cluster areas were also followed-up using a pixel counting image analysis method, which included a cut off at very small clusters formed of cell debris. Cluster size increase measured with either method was larger for primary goldfish keratocyte clusters treated with the Rac1 inhibitor NSC-23766: starting from approximately equal cell numbers as for the untreated co-culture, eventually clusters of lower number and larger size formed. As primary fish keratocyte clusters occupy more overall area, EPC cells of approximately equal number as for the untreated co-culture were here confined to smaller overall area with larger cell density.

**Figure 9:**
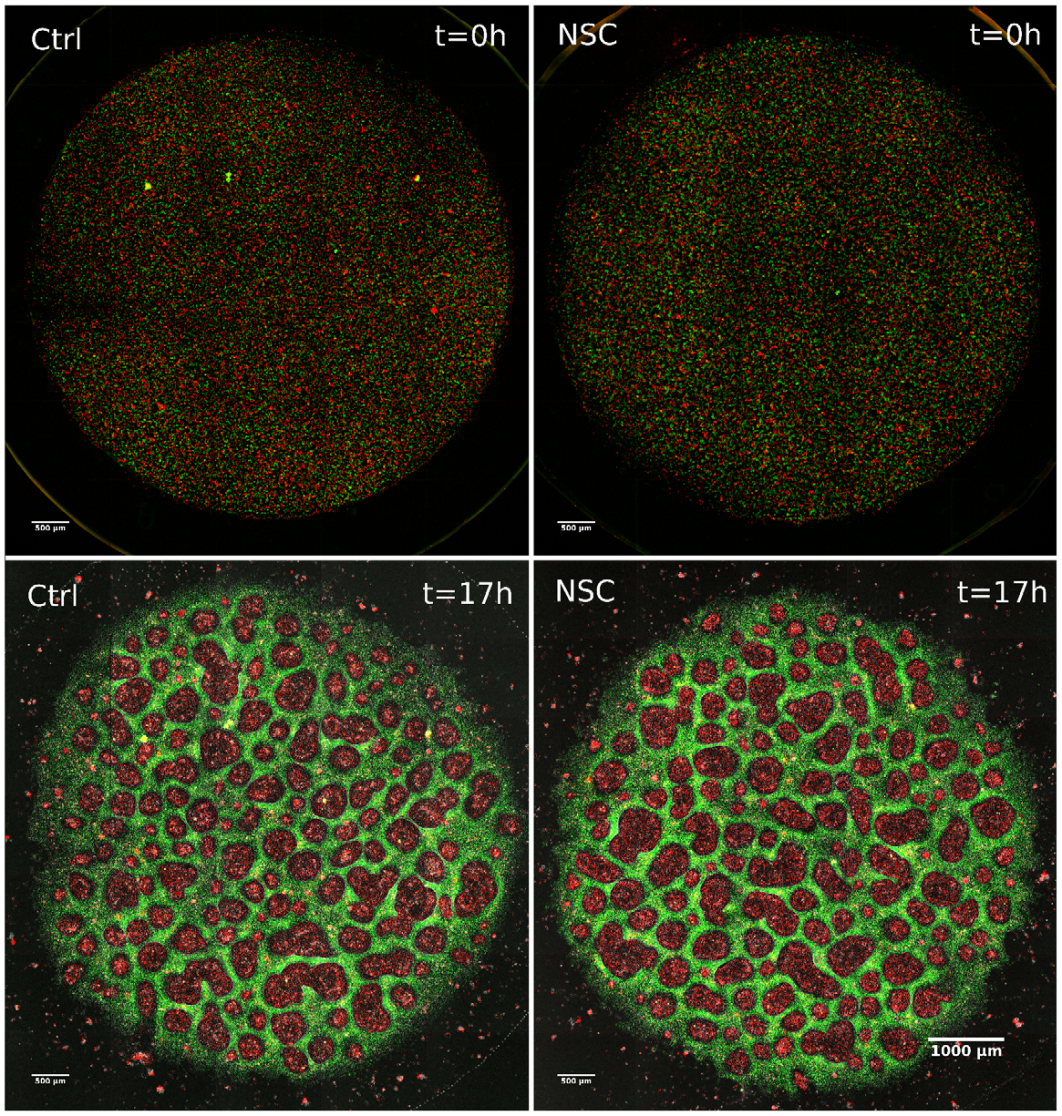
Comparison of cell cluster formation in whole co-cultures without/with Rac1 inhibition. Cluster formation in mixed co-cultures of primary goldfish keratocytes (red) and EPC fish keratocytes (green). Upper panels are initial images of mixed cultures just after cell attachment where equal areas are covered by each cell type (panels A and B). Lower panels show final stage of cluster formation in untreated mixed culture (panel C) and the culture treated with Rac1 inhibitor NSC-23766 (30 µM) from the beginning (panel D). Note the larger cluster sizes of primary goldfish keratocytes (red) in panel C compared to panel D.

**Figure 10:**
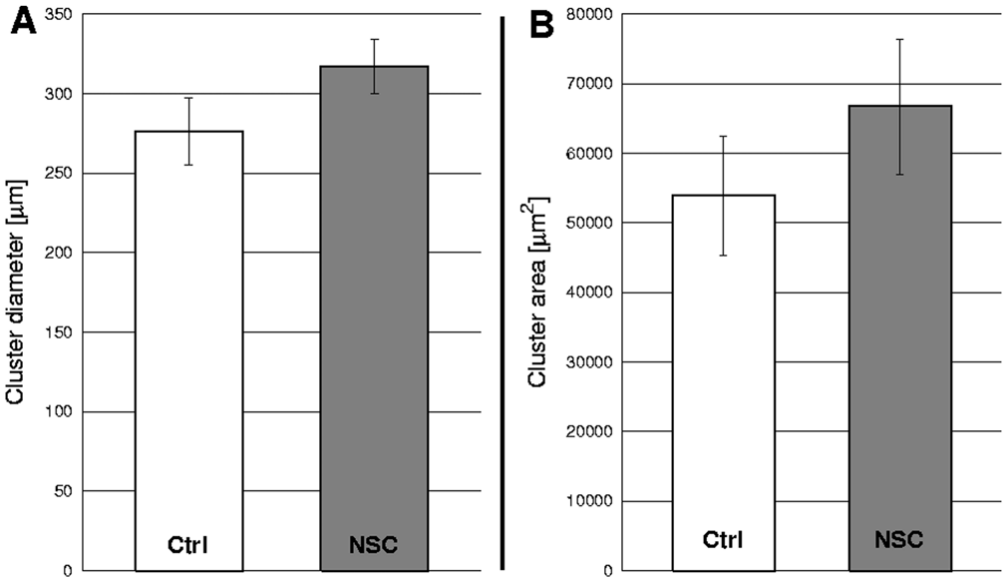
Cell cluster formation data without/with Rac1 inhibition. Comparison of final cluster sizes of primary goldfish keratocytes (PFK) in mixed PFK+EPC co-cultures either untreated (white bars) or treated with Rac1 inhibitor NSC-23766 (30 µM) (grey bars). Cluster diameters, measured as two-point correlation lengths, (panel A) and cluster areas, counted in pixels, (panel B) are compared. Error bars represent SEM, p = 0.03562 for A and p = 0.01238 for B, n = 4 independent experiments.

Several untreated (n = 4) and NSC-treated (n = 4) co-cultures of approx. 200 000 cells covering an area were imaged after cluster formation reached maximum (Figure 9). These images were used for calculations of cluster size and area. Figure 10 shows the comparative data. It is seen that partial Rac1 inhibition and the resultant change in motility leads to formation of larger clusters by primary goldfish keratocytes demonstrated by larger diameter and area values compared to untreated. Paired t-tests were carried out and changes in both diameters and areas were proved to be significant with p-values p = 0.03562 for diameters and p = 0.01238 for areas.

### In vitro cell segregation follows algebraic scaling law

**Figure 11:**
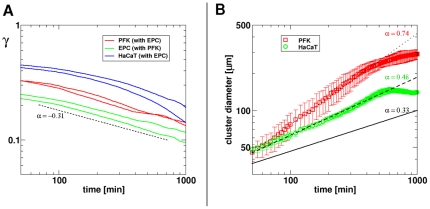
Evolution of the segregation index (γ) and cluster size during cell segregation. Panel A: Segregation index curves of the studied cell types are compared. The value of the segregation index γ indicates the average ratio of other type/all neighbor cells around a given cell calculated for consecutive time points. Note the approximately linear decay of γ on log-log scale beyond 100 minutes in several curves, indicating algebraic scaling. Lines correspond to independent experiments. The slope of the reference dashed line is −0.31. Panel B: Average cluster diameter growth curves calculated from independent experiments with primary fish keratocytes (n = 5) or HaCaT keratocytes (n = 3) are compared. Error bars are standard deviations. The exponent values obtained from fitting straight line segments to the average experimental curves are: 0.74 and 0.48 for PFK and HaCaT, respectively, shown in corresponding colors. Cluster growth curve of simulated segregation of cells without collective motion characterized by exponent value 0.33 is shown for reference (black solid line).

To compare experimental results on the segregation of different cell types with the predicted data of computer simulations [7] we calculated the index indicating the ratio of other type/all neighbor cells around a given cell at a given time point. This index, termed γ, is used for quantifying the degree of segregation. It starts from values determined by the initial mixing ratio of cell types (e.g. γ = 0.5 in case of 1∶1 ratio of mixed cell types) and it decreases over time for every cell type as segregated clusters are formed and grow. In our two-dimensional in vitro experiments the segregation index γ tends not to decrease below 0.1 as the segregation process slows down and halts over ∼1000 minutes (data not shown). The value of γ is proportional to the total length of interface between the segregated cell types. Figure 11 shows the evolution of the segregation index over time with example curves from several independent experiments with the keratocyte types studied. It is apparent that after an initial segment, beyond 100 minutes of segregation, the decay of the segregation index is approximately linear on the log-log scale, with slope values −0.32±0.08 for primary fish keratocytes and −0.35±0.06 for EPC keratocytes, whereas for HaCaT keratocytes the decay is not linear. Linear decay of the segregation index on the log-log scale indicates that the segregation process follows an algebraic scaling law. This finding is in harmony with computer simulations where similar algebraic scaling is predicted for the segregation process in the presence of correlated cell motion, with slope values approx. −0.16 of the decaying γ curves, depending on simulation parameters.

To compare our 2-dimensional in vitro segregation data with earlier 3-dimensional in vitro data [16] we calculated and compared the characteristic growth of cluster sizes. Using the two-point correlation method we calculated the increase in the average diameter *d* of clusters formed by primary fish keratocytes in mixtures with EPC keratocytes and compared with cluster diameters of HaCaT keratocytes in co-culture with EPC keratocytes. These two cell types were chosen as they tend to form isolated clusters (islands) when mixed with EPC keratocytes that tend to form a network.

Figure 11B shows the evolution of cluster sizes during segregation. Data from independent experiments (n = 5 for primary fish keratocytes, n = 3 for HaCaT keratocytes) were averaged and show algebraic scaling behavior in both cases. Lines were fitted to the average experimental diameter growth curves yielding exponents 0.74 and 0.48 for primary fish keratocytes and HaCaT keratocytes, respectively. For comparison, in a simulated system of cells characterized by only diffusive motion with equal and non-equal coverage by two cell types the exponents are 0.33 and 0.25, respectively [8]. The higher exponents describing the characteristic length scale as a function of time in our in vitro system indicate the accelerating effect of the presence collective motion on segregation. Primary fish keratocytes, characterized by the highest directional persistence, yield the highest exponents in the cluster size growth during segregation.

The calculated segregation index γ is inversely proportional to average cluster diameter *d* as segregation evolves. The relationship between 1/γ and *d* is expected to be linear if the forming clusters have identical shape and size at any given time point throughout the segregation process. This, however, does not hold for our in vitro system where cluster diameters are never uniform but have a non-Gaussian, wide distribution with fat tail (data not shown). This can be the reason why the decay of the segregation index and the increase in cluster diameters are characterized by different exponent values.

## Discussion

Pattern formation in various ontogenetic events such as embryonic development involves several driving mechanisms like differential cell adhesion, cell migration and differentiation instructed by diffusible morphogens or mechanochemical interaction of cells with other cells or the extracellular matrix. These mechanisms must act in a concerted or synergistic way in order to provide for robustness of pattern formation, which is considerably resistant to environmental variations.

We demonstrate the existence of an additional patterning mechanism which can speed up and further enhance the robustness of existing and already known mechanisms. This patterning mechanism is based on the different motility characteristics of the pattern forming cell types: cell velocity and directional persistence. It does not require pre-patterned environment and only assumes that adhesion forces between the segregating cell types (heterotypic adhesion) are weak and adhesion forces among cells of their own cell type (homotypic) are strong. If these criteria are met, the speed and extent of cell segregation, measured by the size of segregated homotypic cell clusters, will be strongly influenced by the velocity and ‘collectiveness’ of cell migration, characterized by the directional persistence of migrating cells of each segregating cell type.

Our results from experiments comparing cell types with inherently different collective motility characteristics (Fig. 5) as well as experiments on increasing the directional persistence of one cell type by inhibition of Rac1 GTP-ase (Figs. 8 and 10) support the assumption that cells migrating faster and in a more correlated way with larger directional persistence eventually form larger clusters and more quickly. This way cell segregation becomes faster and more extensive as the collective nature of migration and cell velocity are increased.

Simulations of cell segregation phenomena use cell adhesion, the velocity and correlation of cells' motion and noise as adjustable parameters having considerable impact on the outcome of segregation both in terms of speed and extent [7]. They typically involve a few thousand particles at most. It was shown that even slight increase in the correlation of cells' motion results in considerable acceleration of the segregation process. Without correlation, i.e. with only random cell motion, the time dependence of the segregation process follows a slow logarithmic scaling law, whereas beyond a critical level of local correlation of cells' motion segregation becomes a faster process characterized by algebraic scaling. A newer model also predicts algebraic scaling of the segregation process depending on motile forces of the cell types [17]. Another new simulation model describes the segregation of passive and self-propelled active particles driven only by motility differences and excluded volume interactions, where segregation is enhanced by correlated motion influenced by particle shape [18]. While there are in vitro experiments showing linear growth in segregated cluster size in mixtures of cells from the embryonic neural retina and the pigment epithelium [16], such linear growth is probably not typically characteristic for long-enough processes. Many embryonic developmental processes such as the development of fish embryos exhibit faster growth of emerging patterns than the slow growth of logarithmic scaling predicted by earlier models without correlated cell motion [19], [20], [21], [22]. In our experiments, both the segregated cluster size and the γ index used for quantifying the segregation process evolve showing an algebraic scaling (Fig. 11), which supports the predictions of simulation models including correlated cell motion [7]. Additionally, collective motion strongly increases the growth of cluster sizes, compared to recent data [8] from simulated cell segregation systems without collective motion (Fig. 11B).

Cells' directional migration is based on their inherent ability to maintain their front-rear polarity during migration. Directional migration is influenced by regulators of actin polymerization such as Rac GTP-ase which promotes the network-type polymerization of actin required for both the advance of the cell's lamellopodium at the front and the formation of a new peripheral lamellopodium when the cell changes direction [23], [24]. Slight decrease in Rac activity decreases new peripheral lamellopodium formation leading to an increase in directional persistence [15], which in turn can be interpreted as a decrease in ‘noise’ in simulations. On the one hand, the activity of these actin polymerization regulators are the source of ‘noise’ eventually decreasing directional migration and, on the other hand, they influence the persistence of directional migration which in turn influences correlated cell motion. Directional migration is not independent of adhesion either as clustering and binding to neighbors can increase cells' directional persistence eventually increasing correlated cell motion.

Embryonic development is very fast in several species including the zebrafish where it takes less than 72 hours to complete [25]. During this process extensive cell migrations take place where cell displacements exceed the order of magnitude of individual cell size. Because the instantaneous random motility of cells is in a small range, comparable with cell size, series of random cell displacements driven by differential adhesion could not account for such long range and fast migrations. Such extensive and coordinated cell displacements are thought to be driven at least in part by various external stimuli (e.g. extracellular matrix or morphogens) but given the timing of the process self-propelled collective forms of cell migration also seem to be involved.

Heisenberg and coworkers have recently shown in the developing zebrafish that during epibolic gastrulation the directional persistence and the resultant net speed of mesendodermal cells is regulated by controlling the proportion of different protrusion types: lamellipodium or fillopodium or bleb, formed during migration. Directional persistence is decreased along with an increase in bleb formation, enhanced either by decreasing the membrane to cortex attachment or by increasing the cortical tension of mesendodermal cells. Directional persistence in these cells is regulated independently of instantaneous cell speed [26]. The fine regulation of instantaneous vs. directed cell motility could impact several pattern forming events running along with extensive cell migration in embryonic development.

Computer simulations coupled with and based on experimental findings with live cells could be a very useful tool for advancing our understanding of cell segregation and pattern formation phenomena.

## Materials and Methods

### Cell culture

Cell cultures and co-cultures were made of either freshly isolated primary cells or commercially available cell lines. Cells were kept in Dulbecco's Modified Eagle Medium (DMEM, Sigma) supplemented with either 10% or 15% fetal calf serum (FCS, GIBCO), 4 mM L-glutamine, 100 U/ml penicillin, 0.1 mg/ml streptomycin, 0.25 µg/ml amphotericin B (Sigma) in a humidified incubator with 5% CO2 atmosphere in tissue culture grade Petri dish or 6-well plate (Greiner).

EPC fish keratocyte cell line was grown in DMEM 10% FCS at 25°C, HaCaT human keratocyte cell line and C2C12 mouse myoblast cell line were kept at 37°C in DMEM supplemented with 10% or 15% FCS, respectively. The cell lines were passed after brief incubation with 0.5 mg/ml trypsin and 0.2 mg/ml EDTA (Sigma) in PBS (phosphate buffered saline, 0.1 M phosphate, 0.9% NaCl, pH 7.4, GIBCO).

Primary keratocytes were isolated from scales of goldfish (Carassius auratus). Briefly, without sacrificing the goldfish few of its scales were removed then halved and covered with glass coverslip in DMEM 10% FCS in a tissue culture grade Petri dish at 25°C in a 5% CO2 incubator. Keratocytes migrated out of the scales within one day and subsequently they were passed and seeded after incubating in Ca-free PBS (GIBCO) for 4 minutes then suspending in DMEM 10% FCS.

EPC cell line and primary fish keratocytes were grown at 25°C and imaged at 33°C whereas other cell lines were kept at 37°C.

### Time-lapse microscopy

Time-lapse recordings were performed on either a Zeiss or a Leica system.

Time-lapse recordings with phase contrast and double fluorescence applications were performed on a Zeiss Axio Observer Z1 inverted microscope with 10× EC-Plan Neofluar objective coupled to a Zeiss Axiocam MRM CCD camera and equipped with a Marzhauser SCAN-IM powered stage. For epifluorescent excitation Zeiss Colibri LED illumination system was used. Power stage positioning, focusing, illumination, primary image collection and stitching of primary images into mosaics (2×2 or 5×7) were controlled by Zeiss Axiovision 4.8 software and a custom-made experiment manager software module on a PC.

For time-lapse recordings with phase contrast and single fluorescence applications a computer-controlled Leica DM IRB inverted microscope equipped with a Marzhauser SCAN-IM powered stage was used with a 10× N-PLAN objective (0.25 numerical aperture and 17.6 mm working distance). This microscope was coupled to an Olympus DP70 color CCD camera. Zeiss HXP 120 metal-halide illumination system was used for fluorescent excitation. A time-lapse experiment manager software (CellMovie) controlled the field of view and plane positioning, illumination and image acquisition on a PC.

Cell cultures were kept at 37°C or 33°C in humidified 5% CO2 atmosphere in tissue culture grade Petri dishes (Greiner) in computer-controlled wide-screen microscope stage incubators (CellMovie) mounted on the powered stage of both microscopes.

Phase contrast and epifluorescent images of cells were collected consecutively every 10 minutes from each of the microscopic fields (n = max. 6) for various durations ranging from 12 to 48 hours. Images were edited using NIH ImageJ software.

### Fluorescent cell labeling and imaging

Green and red fluorescent cell tracker dyes were used for labeling and long-term separate imaging of cell types in mixed co-cultures. Membrane labeling green DiO (Invitrogen) was used to label cells excited at 468–475 nm with emissions filtered at 511–542 nm. Cytoplasm staining red CMPTX (Invitrogen) were used with 554–572 nm excitation filter and 597–675 nm emission filter. For double epifluorescence, separate excitations and emissions for either green or red dyes were provided by double band pass filters and double beam splitter arranged in a single filter cube (Zeiss, 74HE filter set), consecutively illuminated at the corresponding wavelengths with separate LEDs by Zeiss Colibri illumination system. The resulting single 8-bit images were subsequently assigned original dye colors and merged using NIH ImageJ software. The illumination system configured this way provided for overlap-free imaging of green DiO and red CMTPX.

For single fluorescence the membrane labeling red DiI (Invitrogen) was used with single band pass excitation (530–560 nm) and emission (572–647 nm) filters (Leica, Y3 filter set).

### Inhibitors

The drug NSC-23766 (Merck) was used in 5–100 µM concentrations in the standard medium to inhibit the small GTP-ase Rac1.

### Data analysis

#### Directional persistence analysis

The motion of individual cells is often evaluated in terms of average cell displacement *d* [27], [28], over a time period *t* as:
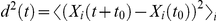
where *X_i_(t)* denoting the center of cell *i* at time *t*, 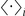 is an average over all possible cells, and *t_0_* is an arbitrary reference frame of the image sequence analyzed. The empirical *d(t)* curves indicate a persistent random walk behavior in monolayer cell cultures studied. Based on the Ornstein-Uhlenbeck model [9] the average displacements are well fitted by Fürth's formula [10]:
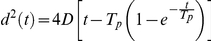
where *T_p_* is the persistence time and *D* is the diffusion coefficient of the long-term random behavior (Tables 1, 2, 3). Thus, for short time periods cells move at a constant speed, termed persistent speed *S*, as the distance is proportional to the time elapsed:
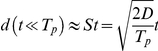
Persistence length, *d_p_*, was calculated from *S* and *T_p_* as 
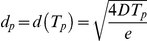
. The fitted parameter values are summarized in Table 1 and show considerable differences for *S*, *T_p_* and *d_p_* depending on the cell type and cell density.

Average cell velocity was also calculated directly from cell displacements in consecutive steps of cell tracking, summarized in Table 1. The velocity, *v_i_(t)*, of a given cell *i* at time *t* was calculated as
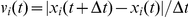
where Δ*t* is the difference of two consecutive steps of cell tracking and thus the time resolution data acquisition. To characterize the motility of an ensemble of cells, time-dependent average velocity *v(t)* was calculated as
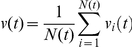
where the summation goes over each *N(t)* cell being in the cell population. Average velocity, *v*, was calculated by averaging *v(t)* over all time steps of cell tracking as
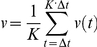
where K is the number of time steps of tracking. Average velocity, *v*, is approximately equal to persistent speed, *S*, if the time resolution of tracking, Δ*t*, is much smaller than persistence time, *T_p_*, however if Δ*t* is comparable with or larger than *T_p_*, the directly measured average velocity is characteristically lower than persistent speed. The error of the manual tracking procedure is estimated to be ∼5 µm, which can also result in differences between the calculated average velocity and persistent speed for cases where Δ*t* is smaller than *T_p_* while cell speed is low.

#### Correlation length analysis

To characterize the average size of clusters, the correlation length of two-point correlations was used. Only the pixels above a threshold were considered. First, we determined the two-point correlations for both colors in a given range (0–200 µm) and calculated the diameter of the clusters. For small distances, the two-point correlation can be approximated as a quadratic form of the distance:
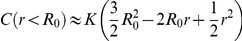
where *R_0_* denotes the average diameter of clusters and *K* is a factor that is usually unknown as it depends on the distribution of the cluster shapes. By fitting a polynomial to the correlations we obtained the typical size of a cluster.

#### Cluster area analysis

We determined the average area of clusters. We used a simple depth-first algorithm to identify clusters and count their size. Since live cell images are generally noisy, simply counting the average size of clusters would result in very small values that could bias the analysis of the effect studied. Furthermore, during the experiments some unviable cells die forming small clusters (5000–10000 µm^2^) that do not participate in the segregation process. For these reasons, we applied a cutoff and we calculated the average area only of those clusters that were larger than 10000 µm^2^.

### Segregation index

We measured the segregation parameter defined by Chaté and coworkers [7]. We divided the images to boxes of 11×11 pixels, corresponding to the average cell sizes, and then we assigned a cell type to each box that occupied the majority of the box area. For the *i^th^* box, we calculated the following quantity:
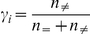
where *n_ = _* is the number of boxes of the same cell type and *n_≠_* is the number of boxes from different cell types around box*_i_*. Finally, we took the average value of this measure for each cell type as the segregation index:
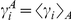
here 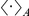 denotes an average over the boxes that are dominated by cells of type *A*.

## Supporting Information

Video S1 **Comparison of motility of cell types studied.** Phase contrast time-lapse videos of primary keratocytes from goldfish (PFK), EPC fish keratocyte line, HaCaT human keratocyte line, C2C12 mouse myoblast cell line.
(MPG)

Video S2 **Density dependent increase in the directional persistence of primary fish keratocytes.** Phase contrast time-lapse video showing low density (left) and high density (right) primary fish keratocyte (PFK) cultures. Note the large increase in the directional persistence of the collectively migrating cell cluster in high density culture compared to single cells.
(MPG)

Video S3 **Density dependent increase in the directional persistence of HaCaT keratocytes.** Phase contrast time-lapse video showing low density (left) and high density (right) HaCaT keratocyte cultures. Note the increase in the directional persistence of cells in the confluent streaming monolayer compared to random migration of single cells at low density.
(MPG)

Video S4 **Spontaneously segregating cell type pairs with low heterotypic adhesion and high homotypic adhesion.** Merged phase contrast + fluorescent time-lapse video showing mixed co-cultures: primary goldfish keratocyte (PFK) + EPC keratocyte (red) (left) and HaCaT + EPC keratocyte (red) (right). Note the growth of homotypic cell clusters in both mixed co-cultures and the formation of the interface between the cell types.
(MPG)

Video S5 **Spontaneously segregating primary fish keratocytes and EPC keratocytes in mixed co-culture.** Merged double fluorescent+phase contrast time-lapse video showing a segregating co-culture of primary goldfish keratocytes (PFK, red) + EPC keratocytes (green). Note the fast growth of homotypic cell clusters.
(MPG)

Video S6 **Spontaneously segregating EPC keratocytes and HaCaT keratocytes in mixed co-culture.** Merged double fluorescent + phase contrast time-lapse video showing a segregating co-culture of EPC keratocytes (red) + HaCaT keratocytes (green). Note the growth of homotypic cell clusters.
(MPG)

Video S7 **Spontaneously segregating HaCaT keratocytes and C2C12 myoblasts in mixed co-culture.** Merged double fluorescent+phase contrast time-lapse video showing a segregating co-culture of HaCaT keratocytes (green) + C2C12 myoblasts (red). Note the slow growth of homotypic cell clusters.
(MPG)

Video S8 **Inhibition of Rac1 enhances spontaneous segregation of primary fish keratocytes and EPC keratocytes in mixed co-culture.** Merged double fluorescent + phase contrast time-lapse video showing a segregating co-culture of primary goldfish keratocytes (PFK, red) + EPC keratocytes (green) not treated (left) or treated with 30 µM NSC-23766 Rac1 inhibitor (right). Note the faster growth of cluster size of primary goldfish keratocytes treated with Rac1 inhibitor.
(MPG)

## References

[1] Turing AM (1952). The chemical basis of morphogenesis, Phil.. Trans R Soc (Lond.).

[2] Murray JD (2003). On the mechanochemical theory of biological pattern formation with application to vasculogenesis.. C R Biol.

[3] Steinberg MS (1963). Reconstruction of tissues by dissociated cells. Some morphogenetic tissue movements and the sorting out of embryonic cells may have a common explanation.. Science.

[4] Steinberg MS (2007). Differential adhesion in morphogenesis: a modern view.. Curr Opin Genet.

[5] Foty RA, Pfleger CM, Forgacs G, Steinberg MS (1996). Surface tensions of embryonic tissues predict their mutual envelopment behavior. Development.

[6] Schötz EM, Burdine RD, Jülicher F, Steinberg MS, Heisenberg CP (2008). Quantitative differences in tissue surface tension influence zebrafish germ layer positioning.. HFSP J.

[7] Belmonte JM, Thomas GL, Brunnet LG, de Almeida RM, Chaté H (2008). Self-propelled particle model for cell-sorting phenomena.. Phys Rev Lett.

[8] Nakajima A, Ishihara S (2011). Surface tensions of embryonic tissues predict their mutual envelopment behavior.. New Journal of Physics.

[9] Ornstein LS (1919). On the Brownian motion.. Proc Amst.

[10] Fürth R (1920). Die Brownsche Bewegung bei Berücksichtigung einer Persistenz der Bewegungsrichtung. Mit Anvendungen auf die Bewegung lebender Infusorien. [in German]. Z Physik.

[11] Szabó B, Szöllösi GJ, Gönci B, Jurányi Z, Selmeczi D (2006). Phase transistion in the collective migration of tissue cells: experiment and model.. Phys Rev E Stat Nonlin Soft Matter Phys.

[12] Szabó A, Unnep R, Méhes E, Twal WO, Argraves WS (2010). Collective cell motion in endothelial monolayers. Phys Biol.. Phys Biol.

[13] Henkes S, Fily Y, Marchetti MC (2011). Active Jamming: Self-propelled soft particles at high density..

[14] Gao Y, Dickerson JB, Guo F, Zheng J, Zheng Y (2004). Rational design and characterization of a Rac GTPase-specific small molecule inhibitor.. Proc Natl Acad Sci U S A.

[15] Pankov R, Endo Y, Even-Ram S, Araki M, Clark K (2005). A Rac switch regulates random versus directionally persistent cell migration.. J Cell Biol.

[16] Beysens DA, Forgacs G, Glazier JA (2000). Cell sorting is analogous to phase ordering in fluids.. Proc Natl Acad Sci U S A.

[17] Kabla AJ (2011). Collective Cell Migration: Leadership, Invasion and Segregation..

[18] McCandlish SR, Baskaran A, Hagan MF (2011). Spontaneous Segregation of Self-Propelled Particles with Different Motilities..

[19] Graner F, Glazier JA (1992). Simulation of biological cell sorting using a two-dimensional extended Potts model.. Phys Rev Lett.

[20] Glazier JA, Graner F (1993). Simulation of the differential adhesion driven rearrangement of biological cells.. Phys Rev E Stat Phys Plasmas Fluids Relat Interdiscip Topics.

[21] Savill NJ, Hogeweg P (1997). Modelling morphogenesis: From single cells to crawling slugs.. J Theor Biol.

[22] Marée AF, Hogeweg P (2001). How amoeboids self-organize into a fruiting body: multicellular coordination in Dictyostelium discoideum.. Proc Natl Acad Sci U S A.

[23] Miki H, Yamaguchi H, Suetsugu S, Takenawa T (2000). IRSp53 is an essential intermediate between Rac and WAVE in the regulation of membrane ruffling.. Nature.

[24] Pollard TD, Blanchoin L, Mullins RD (2000). Molecular mechanisms controlling actin filament dynamics in nonmuscle cells.. Annu Rev Biophys Biomol Struct.

[25] Keller PJ, Schmidt AD, Wittbrodt J, Stelzer EH (2008). Reconstruction of zebrafish early embryonic development by scanned light sheet microscopy.. Science.

[26] Diz-Muñoz A, Krieg M, Bergert M, Ibarlucea-Benitez I, Muller DJ (2010). Control of directed cell migration in vivo by membrane-to-cortex attachment.. PLoS Biol.

[27] Dunn GA, Brown AF (1987). A unified approach to analysing cell motility.. J Cell Sci.

[28] Maheshwari G, Lauffenburger DA (1998). Deconstructing (and reconstructing) cell migration.. Microsc Res Tech.

